# Assessment of Chromosomal Aberrations and S-phase Fraction in Patients With Esophageal Cancer

**DOI:** 10.7759/cureus.84204

**Published:** 2025-05-15

**Authors:** Gaurav Deora, Vasudha Sambyal, Kamlesh Guleria, Jagjeet Kaur, Manjit S Uppal, Meena Sudan

**Affiliations:** 1 Department of Human Genetics, Guru Nanak Dev University, Amritsar, IND; 2 Department of Surgery, Sri Guru Ram Das Institute of Medical Sciences and Research, Amritsar, IND; 3 Department of Radiotherapy, Sri Guru Ram Das Institute of Medical Sciences and Research, Amritsar, IND

**Keywords:** chromosomal instability, esophageal cancer, peripheral blood lymphocytes, s-phase fraction, tumor tissue

## Abstract

Introduction: The varying pace of cell proliferation influences cancer development and persistent growth. S-phase fraction (SPF) refers to the percentage of cells in a tumor in the cell cycle phase, during which DNA is synthesized. SPF has been correlated with nuclear grade, tumor size, estrogen receptor, progesterone receptor, metastasis, and clinical response to neoadjuvant chemotherapy. Aberrations in the number and structure of chromosomes are a common feature of cells from solid tumors. The evaluation of the frequency of chromosomal aberrations in peripheral lymphocytes has been reported as a sensitive cytogenetic assay indicating cancer progression, especially in blood cancer. The present study assessed the correlation between chromosomal aberrations in peripheral blood lymphocytes (PBL) and SPF in the tumor tissue of esophageal cancer patients.

Materials and methods: The present study included 172 subjects, 86 esophageal cancer patients and 86 unrelated age-gender-matched healthy controls. Cytogenetic analysis was done in 172 subjects after 72 hours of peripheral lymphocyte culturing and Giemsa-Trypsin Giemsa banding. SPF was performed in 86 tissue/biopsy samples of esophageal cancer patients using the in vitro bromodeoxyuridine technique.

Results: Chromosomal aberrations were higher in esophageal cancer patients than in healthy controls. The mean (%) aberrant metaphases were significantly higher in patients (35.5 ± 13.3) than in controls (16.0 ± 6.6; p<0.0001). Similarly, mean (%) metaphases with structural aberrations were 16.3 ± 12.0 in patients versus 7.6 ± 5.0 in controls (p<0.0001), while mean (%) metaphases with numerical aberrations were 14.5 ± 7.4 in patients compared to 7.3 ± 4.3 in controls (p<0.0001), and mean (%) metaphases with both structural and numerical aberrations in patients were 4.8 ± 3.5 versus 1.1 ± 1.3 in controls (p<0.0001). Karyotype analysis revealed a higher frequency of chromosomal aberrations, including loss, gain, break, gap, deletion, addition, and translocations, affecting chromosomes 5, 6, 7, 8, 9, 10, 13, 14, 15, 16, 17, 18, 19, 20, 21, 22, X, and Y in esophageal cancer patients. The mean SPF for stage I, stage II, stage III, and stage IV was 8.0±5.0, 8.2±4.8, 8.7 ± 4.2, and 10.8 ± 6.7, respectively. There was no significant difference in the mean SPF among different stages of esophageal cancer patients. However, it was in concordance with the pathological stage of the patients being lowest for the stage I patients and highest for the stage IV patients. The chromosomal aberrations in PBL were also lower in stage I (10%) and higher in stage IV (67%) patients.

Conclusions: Chromosomal aberrations obtained in lymphocytes of esophageal cancer patients showed highly significant differences from those of unrelated healthy controls. The SPF values were higher during the disease progression, with a corresponding high and increasing chromosomal instability in lymphocytes.

## Introduction

In 2022, approximately 20 million new cancer cases and 9.7 million cancer-related deaths were reported globally across both sexes. Nearly half of these new cases (49.2%) and the majority of deaths (56.1%) of cancer deaths occurred in Asia in 2022, which is home to 59.2% of the global population. Corresponding to the incidence burden, the cancer mortality burden in the Asian region is disproportionately greater. This reflects the respective distribution of cancer types alongside comparatively higher case fatality rates on the Asian continent, in part because of late-stage diagnoses. Globally, esophageal cancer in 2022, with 510,716 new cases (364,999 males, 145,717 females) and 445,129 deaths (318,284 males, 126,845 females), ranks 11th in incidence and seventh in mortality, respectively. Incidence and mortality rates of esophageal cancer in India are 97.1 and 66.4 among males and 100.8 and 62.6 among females [1]. Esophageal cancer has two most common histologic subtypes (squamous cell carcinoma and adenocarcinoma), both of which have quite different etiologies, and their incidence varies substantially according to geographical variations. In regions with a high human development index (HDI), smoking and alcohol consumption are the primary risk factors for esophageal squamous cell carcinoma (ESCC). In contrast, the risk factors in low HDI areas remain largely unidentified [2]. In high HDI countries, adenocarcinoma of the esophagus (EAC) accounts for approximately two-thirds of cases. It is strongly linked to factors such as obesity, gastroesophageal reflux disease, and Barrett’s esophagus [3]. Rising rates of adenocarcinoma in several countries suggest that excess body weight may play a significant role in increasing the future burden of esophageal cancer [4,5].

Aberrations in the number and structure of chromosomes are a common feature of cells from solid tumors. There is a causal relationship between chromosomal instability and the initiation or progression of cancer [6]. Centrosomes are crucial in forming bipolar mitotic spindles for accurate chromosomal segregation. Alterations in specific oncogenes and tumor suppressor genes can lead to chromosomal instability by impairing centrosomes' normal function and numerical integrity [7]. Loss of telomere function is an important mechanism for the chromosomal instability commonly found in cancer. Losing a single telomere can result in ongoing instability, affect multiple chromosomes, and generate many rearrangements [8]. In vitro models demonstrate that oncogenic and tumor suppressor pathway deregulation promote and collaborate with chromosomal instability during tumorigenesis [9]. Chromosomal instability often leads to aneuploidy, characterized by an abnormal number of chromosomes in a cell due to errors during cell division [10]. This imbalance can disrupt the normal dosage of oncogenes and tumor suppressor genes. Aneuploidy involving chromosomes that contain genes controlling the mitotic process results in asymmetric segregation of chromosomes, initiating an autocatalytic karyotypic evolution generating preneoplastic and, finally, cancer cells [11,12]. The chromosomal aberrations in lymphocytes could predict human cancer [13]. A 17-year-long follow-up study in a Nordic cohort of 1981 subjects reported a clear association of high levels of chromosomal aberrations with increased total cancer incidence and mortality. The results also suggested greater chances of sporadic cancers than familial ones [14]. Chromosomal instability, although with low frequency, has been reported in PBL of patients affected by some types of cancers, such as retinoblastoma [15-17], familial polyposis of the colon [18], Hodgkin’s disease [19], breast carcinoma [20,21], and multiple endocrine neoplasia type 1 [22]. A decrease in the frequency of chromosomal aberrations after tumor removal has also been observed. [20]. The evaluation of the frequency of chromosomal aberrations in peripheral lymphocytes has been reported as a sensitive cytogenetic assay for detecting exposure to mutagens and carcinogens [23]. Genomic aberrations lead to gene dysfunction, carcinogenesis, and tumor progression [24]. There is a high frequency of chromosomally relevant genomic alterations in EAC and ESCC [25]. High frequency of loss of heterozygosity was reported in ESCC [26,27]. Copy number variations were thought to have an association with ESCC, where an increase in copy number in chromosome 3q26 was a common and early event in ESCC [28,29].

The varying pace of cell proliferation influences the development and persistent growth of cancers [30]. SPF refers to the percentage of cells in a tumor that are in the phase of the cell cycle during which DNA is synthesized. SPF reveals cell proliferation by assessing the percentage of a particular cycling subpopulation between the G0/G1 and G2/M phases [31]. SPF represents the proportion of cells preparing for mitosis by their active replication of the DNA content [32], and its evaluation has become one of the typical methods of determining proliferation [30]. A high SPF means that the cancer cells divide more rapidly and tend to be more aggressive. Prognostic factors help to assess the therapeutic requirements. Survival of myeloma patients has been observed to vary according to the growth rate of tumor cells [33]. Tumor growth can be assessed through proliferation kinetics using the labeling index, which indicates the proportion of plasma cells in the S-phase relative to the total number of plasma cells counted [33]. A labeling index exceeding 1% has been linked to poor survival outcomes [34-36]. SPF's prognostic value was compared to the Ki-67 index in breast carcinoma [37]. SPF was found to be a better prognosticator than the Ki-67 index. High SPF is independently associated with a worse clinical outcome for patients with breast cancer [38]. SPF is a valuable predictor of survival and can confidently be assessed in approximately 80% of cases [39]. Given its association with mitotic activity, the SPF should be considered a valuable prognostic marker for routine use in the clinical management of breast cancer [39]. High SPF has been extensively acknowledged as a considerable indicator for breast cancer survival [40-42], and its correlation with nuclear grade, tumor size, ER, PR, lymph node metastasis, and age has been shown [40,43,44].

The present study was designed to analyze chromosomal aberrations in PBL of esophageal cancer patients and the SPF in the tumor tissue of esophageal cancer patients to prospectively explore their prognostic/diagnostic significance with increased sensitivity and specificity that could help in identifying the rapid progression of cancer. Chromosomal aberrations in PBL of esophageal cancer patients were examined, and the BrDU labeling technique was used to assess the SPF in the esophageal tumor tissue. The chromosomal aberrations were also assessed for the unrelated healthy controls and were compared to those of esophageal cancer patients.

## Materials and methods

Study subjects

In the present study, 172 subjects were recruited from Amritsar, Punjab, North-West India. Clinically diagnosed esophageal cancer patients were recruited from Sri Guru Ram Das Institute of Medical Sciences and Research, Vallah, Amritsar. The study was conducted following ethical clearance from the Institutional Ethics Committee of Guru Nanak Dev University, Amritsar, Punjab, India (approval number: 300/HG, approval date: 07-04-2022). Patients who had received chemotherapy, radiotherapy, or blood transfusion before surgery or had a previous history of any malignancy were excluded from the study. Annexure I presents the detailed inclusion/exclusion criteria for patients and controls, ensuring methodological transparency and consistency. Upon obtaining informed consent, detailed epidemiological information was gathered from each participant using a pre-tested structured questionnaire covering age, gender, occupation, personal history, habitat, habits, medical history, etc. Tissue/biopsy samples of 86 patients were collected in a vial containing culture medium (RPMI 1640).

SPF analysis

SPF was carried out using the in vitro BrDU technique proposed by Hemmer [45] with slight modifications. Antibody labeling was performed, and cells in S-phase were scored under a fluorescent microscope. SPF was assessed in 1000 cells for each subject. Refer to the detailed protocol in Annexure III for a comprehensive and standardized approach to SPF assessment.

Cytogenetic analysis

To investigate cytogenetic abnormalities, blood samples were obtained from 86 esophageal cancer patients and 86 age- and gender-matched healthy controls with no family history of cancer. Samples were collected in a heparinized vial, and lymphocyte cultures were established using the standard 72-hour protocol using phytohemagglutinin as a mitogen. GTG banding was performed, and karyotyping was conducted in accordance with the International System for Human Cytogenomic Nomenclature (ISCN) 2016 guidelines [46]. Annexure II provides detailed, step-by-step protocols for PBL culturing, harvesting, and GTG banding to facilitate accurate and reproducible results. Chromosomal aberrations were evaluated by analyzing 50 to 100 metaphases per individual and classified into total aberrant metaphases (TAM), metaphases with structural aberrations (MSA), metaphases with numerical aberrations (MNA), and those exhibiting both structural and numerical aberrations (M(NA + SA)). A comparison of cytogenetic aberrations observed in esophageal cancer patients and control subjects was done to identify anomalies associated with esophageal cancer.

Statistical analysis

The statistical analysis was done to correlate the S-phase values with the pathological stage of the patient. The continuous independent variables were expressed as mean ± standard deviation using the Student’s t-test. This test was also used to demonstrate the significant difference in S-phase values among different stages of esophageal cancer patients. Student’s t-test was used to compare the frequency of chromosomal aberrations in cases and controls. The ORs, 95% CI ranges, and corresponding p-values were calculated to measure the relative risk or strength of association. Statistical significance was determined at p≤0.05, and all the analyses were carried out using SPSS Statistics version 16 (SPSS Inc. Released 2007. SPSS for Windows, Version 16.0. Chicago, SPSS Inc.).

## Results

Demographic and clinical details of study participants

The study included 86 patients with pathologically confirmed esophageal cancer (59.3% females and 40.7% males) and 86 healthy controls. The characteristics of esophageal cancer patients and controls are summarized in Table 1. The mean age of esophageal cancer patients was 58.8 ± 13.0 years (range 28-84 years) and that of controls was 55.2 ± 13.0 years (range 30-85 years). Esophageal cancer incidence was higher among individuals more than 50 years of age (81.4%) compared to those less than 50 years (18.6%). Among the 86 cancer patients, 8.1% were diagnosed with stage I, 33.7% with stage II, 22.1% with stage III, and 8.1% with stage IV esophageal cancer. There were 24 patients whose stage couldn't be determined accurately from early biopsy, as they didn't turn up for further testing or treatment. No significant differences were observed between the esophageal cancer patients and the control group in terms of gender, age, habitat, diet, alcohol consumption, and menstrual history (p>0.05). However, there was a significant difference in the smoking habits of the esophageal cancer patients and controls (p=0.01) (Table 1).

**Table 1:** Clinical characteristics of esophageal cancer patients and controls * business, laborer, driver, employee Alcoholics, smokers, and alcoholics + smokers were all males, statistically significant p-values are displayed in bold OR: odds ratio, CI: confidence interval, SD: standard deviation

Variable	Patients	Controls	OR (95% CI)	p-value
	n (%)	n (%)	-	-
Gender	-	-	-	-
Male	35 (40.7)	35 (40.7)	-	-
Female	51 (59.3)	51 (59.3)	-	-
Age (year)	-	-	-	-
<50	16 (18.6)	25 (29.0)	Reference	-
≥50	70 (81.4)	61 (71.0)	1.79 (0.88-3.67)	0.1096
Mean ± SD	58.8 ± 13.0	55.2 ± 13.0	-	-
Range	28-84	30-85	-	-
Habitat	-	-	-	-
Rural	65 (75.6)	65 (75.6)	-	-
Urban	21 (24.4)	21 (24.4)	-	-
Habits	-	-	-	-
Diet	-	-	-	-
Vegetarian	41 (47.7)	50 (58.1)	Reference	-
Non-vegetarian	45 (52.3)	36 (41.9)	1.52 (0.83-2.78)	0.17
Alcohol drinking	-	-	-	-
Never	57 (66.3)	67 (77.4)	Reference	-
Ever	29 (33.7)	19 (22.6)	1.79 (0.91-3.53)	0.09
Smoking status	-	-	-	-
Never	73 (84.9)	83 (96.8)	Reference	-
Ever	13 (15.1)	3 (3.2)	4.93 (1.35-17.98)	0.01
Alcoholic + smokers	9 (10.5)	3 (3.2)	-	-
Occupation	-	-	-	-
Farmers	13 (15.1)	15 (17.7)	-	-
Housewives	44 (51.2)	50 (58.1)	-	-
*Others	29 (33.7)	21 (24.2)	-	-
Menstrual status	-	-	-	-
Pre-menopausal	10 (19.6)	17 (32.5)	Reference	-
Post-menopausal	41 (80.4)	34 (67.5)	2.05 (0.83-5.06)	0.12

Cytogenetic analysis

Cytogenetic analysis was performed on 86 esophageal cancer patients and 86 age- and gender-matched controls. The majority of the patients (96.5%) had squamous cell carcinoma. The chromosomal aberrations were counted as in MSA, MNA, and M(SA + NA). The difference in the frequencies of chromosomal aberrations amongst patients and controls was statistically significant (Table 2). The aberrations were higher in patients as compared to controls: mean (%) aberrant metaphases (35.5 ± 13.3 vs. 16.0 ± 6.6; p<0.0001), mean (%) MSA (16.3 ± 12.0 vs. 7.6 ± 5.0; p<0.0001), mean (%) MNA (14.5 ± 7.4 vs. 7.3 ± 4.3; p<0.0001), mean (%) M(SA + NA) (4.8 ± 3.5 vs. 1.1 ± 1.3; p<0.0001). Compared to controls, patients had two-fold higher frequencies for all categories of aberrations.

**Table 2:** Cytogenetic profile of esophageal cancer patients and controls Significant p-values (<0.05), calculated by t-test, are shown in bold MSA: metaphases with structural aberrations, MNA: metaphases with numerical aberrations, M(SA + NA): metaphases with structural and numerical aberrations, SD: standard deviation

	Patients	Controls	p-value
No. of subjects	86	86	-
Age (mean ± SD)	58.8 ± 13.0	55.2 ± 13.0	0.0711
Mean (%) aberrant metaphases	35.5 ± 13.3	16.0 ± 6.6	<0.0001
Mean (%) MSA	16.3 ± 12.0	7.6 ± 5.0	<0.0001
Mean (%) MNA	14.5 ± 7.4	7.3 ± 4.3	<0.0001
Mean (%) M(SA + NA)	4.8 ± 3.5	1.1 ± 1.3	<0.0001

The stage-wise comparison of the cytogenetic profile of esophageal cancer patients with controls (Table 3) showed higher frequencies of different types of chromosomal aberrations in patients as compared to controls. Compared to controls, a twofold increase was observed in the frequency of TAM in stage III and total MSA in stage IV patients. The frequency of TAM and total MSA in stage II was threefold higher than in controls. The frequency of TAM showed a higher frequency in stage II (42.3 ± 13.1), followed by stage III (34.7 ± 13.4), stage IV (32.7 ± 9.3), and stage I (24.6 ± 7.0). The frequency of total MSA showed a higher frequency in stage II patients (21.3 ± 14.8), followed by stage IV (16.0 ± 11.2), stage III (14.8 ± 9.1), and stage I (12.9 ± 9.8). The frequency of total MNA was higher in stage III (16.0 ± 8.6), followed by stage II (14.4 ± 6.4), stage IV (12.3 ± 5.2), and stage I (9.9 ± 4.0). The patient group whose stage couldn't be determined also showed higher frequencies of different chromosomal aberrations than controls. When total metaphases having both structural and numerical aberrations together were compared among the stages, stage II (6.6 ± 4.3) showed the highest frequency, followed by stage IV (4.6 ± 2.6), stage III (3.9 ± 2.9), and stage I (1.8 ± 1.1).

**Table 3:** Stage-wise comparison of cytogenetic profile of esophageal cancer patients and matched controls and mean SPF Significant p-values (<0.05), calculated by t-test, are shown in bold TAM: total aberrant metaphases, MSA: metaphases with structural aberrations, MNA: metaphases with numerical aberrations, M(SA + NA): metaphases with structural and numerical aberrations, SPF: S-phase fraction

Patient group and controls	Mean (%) TAM	Mean (%) MSA	Mean (%) MNA	Mean (%) M(SA + NA)	Mean SPF
Stage I cases (n=7)	24.6 ± 7.0	12.9 ± 9.8	9.9 ± 4.0	1.8 ± 1.1	8.0 ± 5.0
Controls	16.0 ± 8.5	8.5 ± 5.6	6.9 ± 2.5	0.6 ± 0.9	-
p-value	0.0611	0.3227	0.1182	0.0453	-
Stage II cases (n=29)	42.3 ± 13.1	21.3 ± 14.8	14.4 ± 6.4	6.6 ± 4.3	8.2 ± 4.8
Controls	16.8 ± 6.0	7.7 ± 3.8	8.0 ± 4.6	1.1 ± 1.2	-
p-value	<0.0001	<0.0001	<0.0001	<0.0001	-
Stage III cases (n=19)	34.7 ± 13.4	14.8 ± 9.1	16.0 ± 8.6	3.9 ± 2.9	8.7 ± 4.2
Controls	15.1 ± 5.5	8.0 ± 5.3	5.8 ± 2.9	1.3 ± 1.6	-
p-value	<0.0001	0.0079	<0.0001	0.0016	-
Stage IV cases (n=7)	32.7 ± 9.3	16.0 ± 11.2	12.3 ± 5.2	4.6 ± 2.6	10.8 ± 6.7
Controls	16.8 ± 9.5	8.5 ± 9.1	7.7 ± 4.9	0.6 ± 0.8	-
p-value	0.0082	0.1942	0.1142	0.0021	-
Indeterminate stage cases (n=24)	29.0 ± 11.5	9.2 ± 5.7	16.1 ± 9.8	3.8 ± 1.7	9.9 ± 5.0
Controls	14.8 ± 7.7	5.6 ± 3.0	8.0 ± 6.1	1.1 ± 1.3	-
p-value	<0.0001	0.0088	0.0013	<0.0001	-

Further, mean (%) TAM, mean (%) MSA, mean (%) MNA, and mean (%) M(NA + SA) were compared among different stages of esophageal cancer patients. A significant difference was observed in the mean (%) TAM among stage I and stage II (p=0.0016) and the mean M(NA + SA) among stage I and stage II (p=0.0065), stage I and stage IV (p=0.0222), and stage II and stage III (p=0.0206).

Table 4 shows the chromosomes frequently involved in aberrations like loss, gain, deletion, addition, and translocations. The control subjects had predominantly normal karyotypes, and the chromosomal aberrations found were less frequent than in cases. Moreover, no specific or recurring anomaly was observed in controls.

**Table 4:** Chromosomes involved in various aberrations in esophageal cancer patients and controls

Type of aberration	Patients	Controls
Loss	6,7,8,10,13,15,16,17,18,19,20,21,22,X,Y	8,9,10,15,16,18,20,22,X,Y
Gain	4,5,8,10,13,14,18,20,21,22,X	3,6,7,9,10,12,16,19,20,22
Break (chromosome and chromatid)	1,2,4,5,7,9,12	1,2,3,4,5,9,16
Gap (chromosome and chromatid)	2,3,4,14,16,X	1,2,5,14,17
Deletion	1, 2, 3, 4, 5,11,14,15,X	1,5,6,X
Addition	1, 9	9
Translocations	7,14,X	2,4,16
Robertsonian translocation	13,14,15, 21,22	13,14,21,22
Telomeric associations	1,2,3,4,5,12,13,14,15,16,17,18,19,20,21,22,Y	7,13,14,15,19,21,22,X
Triradials	13,14,15,21,22	14,15,21,22
Ring	8	-

SPF analysis

**Figure 1:**
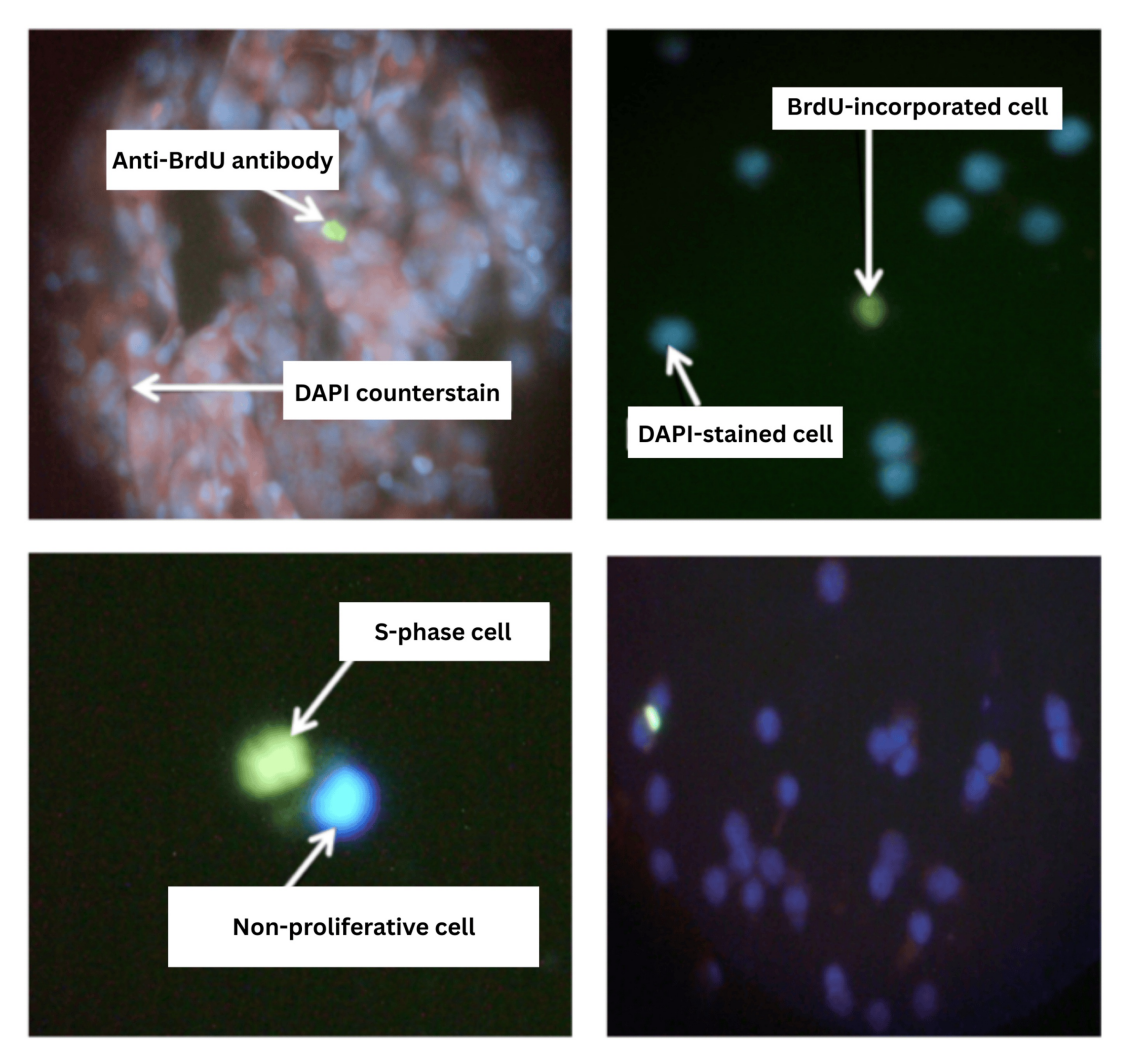
Cells labelled with anti-BrdU antibody and counterstained with DAPI BrdU: bromodeoxyuridine, DAPI: 4',6-diamidino-2-phenylindole

Figure 1 shows cells labelled with anti-BrdU antibody and counterstained with 4',6-diamidino-2-phenylindole (DAPI). Stage-wise mean SPF of esophageal cancer patients has been shown in Table 3. The mean SPF was highest in stage IV (10.8 ± 6.7) patients, followed by stage III (8.7 ± 4.2), stage II (8.2 ± 4.8), and stage I (8.0 ± 5.0). The range of SPF for stage I cancer patients was 2.0-16.7; for stage II cancer patients, the range was 1.7-28.7; for stage III cancer patients, the range was 2.1-16.3; and for stage IV cancer patients, the range was 1.3-19.4. Overall, SPF varied from 1.2 to 28.7. There was no significant difference in the mean SPF among different stages of esophageal cancer patients. However, it was in concordance with the pathological stage of the patients being lowest for the stage I patients and highest for the stage IV patients. The mean SPF of a patient group whose stage couldn't be determined was 9.9 ± 5.0, greater than the mean SPF of stage I, stage II, and stage III esophageal cancer patients. The chromosomal aberrations in PBL were also lower in stage I (10%) and higher in stage IV (67%) patients.

## Discussion

Chromosomal instability is an enhanced frequency of chromosome missegregation due to errors in the cell cycle [47,48]. Several epidemiological studies have reported the chromosomal aberration frequency in PBL to be associated with higher cancer risk [49]. Chromosome instability is the main feature of most cancers. Aneuploidy, caused by unbalanced division of chromosomes during cell division, occurs frequently in many solid tumors [50].

In the present study, the mean frequency of aberrant metaphases, structural aberrations, numerical aberrations, and structural and numerical aberrations was higher in esophageal cancer patients than in controls (p<0.0001). In esophageal cancer patients, loss of chromosomes 6, 7, 8, 10, 13, 15, 16, 17, 18, 19, 20, 21, 22, X, and Y, and gain of chromosomes 4, 5, 8, 10, 13, 14, 18, 20, 21, 22, and X were more frequent among the numerical aberrations. The loss of chromosomes 6, 7, 13, 17, 19, and 21 and the gain of chromosomes 4, 5, 8, 13, 14, 18, and 21 were typical of the esophageal cancer patient compared to the controls. Loss of chromosomes 3, 8, 10, 20, and Y in ESCC patients has been reported earlier in a study based in China [51].

The present study observed repeated structural chromosomal aberrations, breaks, and gaps in esophageal cancer patients. Breaks were frequently observed on chromosomes 1, 2, 4, 5, 7, 9, and 12. Breaks on chromosomes 7 and 12 and gaps on chromosomes 3, 4, 16, and X were exclusive to esophageal cancer patients as compared to controls. Multiple chromosomal loci have been reported to be involved in the development and progression of esophageal cancer [52]. Chromosome 1 was found to be involved in different structural aberrations in the present study. In esophageal cancer, loss of expression of p73 and N-ras oncogene mutations on chromosome 1 has been associated with the development of cancers. The tumor suppressor genes present on chromosome 1 are NRAS (1p13.2), MYCL1 (1p34.2), BCL10 (1p22), GDB2 (1p34.3), MUTYH (1p32.1), and SK1 (1q22) (http://atlasgeneticsoncology.org/). Structural alterations in chromosomes 3, 4, 5, 6, 8, 9, 10, 11, 12, 17, and 19 are associated with bladder cancer risk. Chromosomes 4, 8, and 9 are significantly associated with the risk of both adenocarcinoma and squamous cell carcinoma [53].

The manifestation of unstable chromosomes as premature centromere division, fragile sites, telomeric associations, breaks, and gaps was observed in the present study. Premature centromeric division was also frequently observed in the cases in the present study. Premature centromere division is manifested as a loss of control of centromere separation and segregation and is characterized by distinctive separation of chromatids earlier than usual during interphase of mitosis [54]. A study reported fragile sites commonly stable in cultured cells but highly susceptible to chromosomal deletions, rearrangements, and sister chromatid [55].

In the current study, the frequency of telomeric associations in esophageal cancer patients was higher than in controls. Studies have reported telomeric associations with various human tumors [56]. A strong association exists in lung, stomach, esophagus, liver, and prostate cancers. Telomeric associations between chromosomes 15pter and 20qter and between chromosomes 1q and 22qter evolved stepwise to ring chromosomes 20 and 22 [57].

In the present study, the frequency of Robertsonian translocations was higher in esophageal cancer patients than in controls. Robertsonian translocations between chromosomes 21;22, 13;21, and 14;21 were more common in the present study than Robertsonian translocations between other acrocentrics. Compared to controls, Robertsonian translocations involving chromosome 15 were present only in esophageal cancer patients. It has been suggested in previous reports that a higher frequency of rob(13;14) and rob(14;21) in comparison to other Robertsonian translocations might be due to the homologous but inverted segments present in chromosomes 13, 14, and 21, resulting in more frequent crossover and recombination [58,59].

The proliferation level of tumors is an important predictor of survival in cancer. Proliferation is also reported as the common driving force for several promising prognostic gene expression signatures [60]. Different parameters like Ki-67 immunohistochemistry (IHC), histological grade (HG), and mitotic activity index (MAI) have been reported as classical methods of proliferation level [61]. However, the prognostic implications of these parameters are still unclear [62]. Recently, a study reported a genomic grade index based on a 128-probe expression profile as a promising prognostic indicator in patients with or without adjuvant systemic therapy, which outperformed the HG, MAI, and Ki-67 IHC parameters [63].

Prognostic value of SPF and linking of clinicopathological variables, Ki-67 and SPF, with disease outcome were also reported [64]. Low Ki-67 and SPF have been associated with a low rate of disease occurrence and longer survival time. Between Ki-67 and SPF, the latter showed a more significant association with a high-grade malignancy. In the present study, the S-phase was assessed for esophageal cancer patients. The value of SPF ranged from 1.2 to 28.7 and corresponded well with the cancer stage. The highest mean SPF was observed in patients at stage IV (10.8 ± 6.7), and the lowest mean SPF was observed in patients at stage I (8.0 ± 5.0). A high value of SPF indicated more aggressive tumor cells with poor prognosis, as a strong correlation had been reported between high SPF and increased risk of recurrence and mortality in patients with carcinomas [65]. In the present study, the mean SPF of a patient group whose stage couldn't be determined was 9.9 ± 5.0 and was greater than the mean SPF of stage I, stage II, and stage III esophageal cancer patients, depicting their aggressiveness.

Though there has been criticism concerning SPF values due to its inability to distinguish cells that were actively synthesizing DNA from the putative S-quiescent cells [66], SPF has been considered to be the most useful cell proliferation method in predicting the short-term prognosis of patients with cancer [37], with the conventional median SPF category being the best indicator of disease outcome compared with other SPF variables and the Ki-67 index. In the present study, 24 patients with stage I and II cancer have a low value of SPF (1.7-3.9). Early-stage cancer patients with low SPF have shown an association with better survival [67]. The prognostic relevance of SPF measurement has been reported in predicting the long-term overall survival of patients with early-stage or locally advanced cancers [68]. Eight patients with stage III cancer thus had a better prognosis, as their SPF values were low (2.1-5.4). Ploidy and, in particular, SPF had been addressed as rather powerful prognostic factors in node-negative breast cancer [69]. SPF in breast cancer had a significant prognostic impact on disease-free survival in node-negative patients [70]. DNA ploidy and SPF were reported as useful biomarkers for managing patients with invasive breast cancer. They are only marginally inferior to axillary lymph node status in their relative importance in predicting disease outcome [71].

In conjunction with mitotic activity, SPF could become a prognostic factor that could be used in daily practice by oncologists for the management of carcinomas [72]. SPF was a robust predictor of clinical outcome in terms of overall survival, relapse-free survival, and survival after relapse [73]. SPF analysis might be important in sequentially analyzing tumor cell proliferation under chemo- or radiotherapy [74,75]. SPF estimated by flow cytometry (FCM) and computed from DNA histograms was reported as a significant prognostic factor and a good predictor of response to neoadjuvant chemotherapy [76,77]. A change from diploid to aneuploid or a rise in SPF might reveal a failure in therapy even before changes in tumor size become evident. Modifying the proliferative rate in response to treatment was proposed as a prognostic marker [78,79]. In the present study, the SPF values by the BrDU method were higher during the disease's progression. Hence, SPF might act as an important prognostic marker to study the progression and aggressiveness of the disease and later recession of the disease post-therapy. In the present study, the patients who didn't turn up for further testing or treatment corresponded well with the disproportionately greater mortality concerning incidence in the Punjab region [1]. The mean SPF of this patient group was greater than the mean SPF of stage I, stage II, and stage III esophageal cancer patients, depicting their aggressiveness. For such patients, their SPF values could be used to prevent late-stage diagnoses and subsequent higher case fatality rates in lower HDI settings.

Strengths and limitations of the study

Classical cytogenetic analysis is considered the best tool for obtaining an overall picture of the type and frequency of chromosomal changes in cancer. Chromosomal aberrations could also be used for screening, as they are the only noninvasive method available. In the current study, SPF was evaluated by fluorescent microscopy as per Hemmer's protocol [45]. Compared to the previous results reported using FCM, which is an expensive technique and requires skilled technicians, the present technique is simple and comparatively less expensive.

Limitations: The strengths of the present study also act as its limitations. A cytogenetic approach has been used in the present study to identify a high mean frequency of structural aberrations in esophageal cancer patients compared to healthy control individuals. The present study was limited by using GTG banding for cytogenetic analysis. G-banding has a lower resolution than other microscopy-based alternatives [80]. In the present study, SPF was determined using fluorescent microscopy; in contrast, the most studied and accessible method for evaluating proliferative activity was the determination of SPF using FCM, which was shown to be a reliable and reproducible method [81]. Finally, the present study was limited by its relatively small sample size, which may affect the generalizability of the findings and warrants validation in larger cohorts.

## Conclusions

In the present study, conventional cytogenetics and SPF results were studied in the context of esophageal cancer patients. Statistical analysis showed highly significant differences in chromosomal aberrations obtained in lymphocytes of esophageal cancer patients compared to those of unrelated healthy controls. There was an overall increase in aberrations in stage II patients compared to patients at stage I. S-phase values can measure cell proliferation in esophageal cancer by fluorescent microscopy, which is simple and less expensive. In the current study, the SPF values were higher during the disease progression, corresponding to high lymphocyte chromosomal instability. Chromosomal aberrations in PBL thus appear to be related to proliferating tumors. SPF and lymphocytic instability could therefore be used as prognostic markers to study the progression of cancers. The present study depicted that in lower HDI settings, SPF values could prevent late-stage diagnoses and higher mortality in this region. Measurement of SPF, along with conventional cytogenetics, does have clinical utility for patients with esophageal cancer and could be used as a better prognostic factor. Monitoring the changes in SPF in patients on therapy might enable the physician to select which patients are more likely to obtain a complete response or no response to primary treatment. However, standardization and quality control must be improved before they can be routinely used as adjuncts to classical parameters.

## Appendices

Annexure I

Selection Criteria for Patients and Controls

I.I. Inclusion Criteria for Patients

1. Preoperative patients with histopathology-confirmed esophageal cancer

2. Without a prior history of any cancer

I.II. Exclusion Criteria for Patients

1. Patients who had received chemotherapy, radiotherapy, or undergone surgery.

2. Blood transfusion less than three months from the date of collection of the blood sample

II.I. Inclusion Criteria for Controls

1. Age and gender matched, unrelated healthy individuals, residents of the same geographical area as patients.

2. No history of any cancer or sign of any other malignancy or chronic disease.

II.II. Exclusion Criteria for Controls

1. Individuals suffering from any chronic or malignant disease 

2. On regular medication

3. Family history of any cancer

Annexure II

Peripheral Blood Lymphocyte Culturing, Harvesting, and G-banding Protocols

I. Setting of Lymphocyte Culture

1. Four ml of blood was drawn intravenously using a disposable syringe with a 22G needle and transferred to a heparinized vacutainer.

2. Blood was allowed to settle for three to four hours, so plasma separated as a top layer of straw color liquid. A thin white layer of leucocytes (buffy coat) appeared at the junction of plasma and RBCs.

3. Prior to setting the culture, the UV light of the laminar flow unit was switched on for 10 minutes. Culture vials, caps, and syringes were kept under UV light.

4. Eight millilitres of RPMI-1640 (Himedia) media culture were added to the autoclaved culture vial.

5. Then, 1.5 ml fetal bovine serum (FBS) aliquots were added to each culture vial containing the RPMI-1640 culture media.

6. The plasma and the buffy coat were aspirated using a spinal needle and put in the culture vial containing media. A few drops of RBCs were also added.

7. Finally, 0.15 ml of PHA-M was added to the culture vial, and the time was noted.

8. The culture vial's mouth was flamed and tightly closed with a cap. The contents were well mixed by gently shaking.

9. The culture vial was kept in an incubator and incubated at 37°C for 72 hours. It was agitated twice daily to ensure proper mixing and growth during this period.

10. About 0.05 ml of demecolcine (Himedia) was added to the culture vial one hour prior to cell harvesting and mixed gently. The culture was terminated after one more hour of incubation at 37°C.

II. Harvesting of Culture

1. After 72 hours, the contents of the culture vial were transferred into an autoclaved centrifuge tube and spun at 800 rpm for 10 minutes.

2. The supernatant was discarded, leaving some liquid behind. During the procedure, care was taken not to disturb the cell pellet at the bottom.

3. To resuspend the pellet, it was disturbed by tapping the side of the centrifuge tube.

4. A few drops of freshly prepared hypotonic solution pre-warmed (37°C) were slowly added along the side of the centrifuge tube, accompanied by gentle shaking, and the final volume was raised to 8 ml.

5. The tube was incubated at 37°C for 17 minutes. After incubation, the contents were spun at 800 rpm for 10 minutes, and the supernatant was discarded.

6. Gentle tapping disturbed the pellet. Eight milliliters of freshly prepared chilled fixative (3:1 methanol/acetic acid) were added along the wall of the tube, followed by gentle pipetting. One milliliter was added first. As the solution turned dark in color, it was appropriately shaken. Subsequently, the tube was filled with fixative to 8 ml.

7. The centrifuge tube was kept at 4°C in the refrigerator for 30 minutes. After 30 minutes, the contents were spun at 800 rpm for 10 minutes, and the supernatant was discarded.

8. The pellet was disturbed by gentle tapping, and 8 mL of fresh fixative was added. The suspension was kept at 4°C for a few hours or until slide preparation

III. Slide Preparation

1. On the day of slide preparation, the cell suspension was removed and spun at 800 rpm for 10 minutes. The supernatant was again discarded, and the pellet was retained, well mixed, and 8 ml of fresh fixative was added.

2. Three to four similar washings of fixative were given until a clear pellet was obtained. The pellet was correctly mixed by gentle pipetting.

3. Precleaned and prelabelled slides were thoroughly washed and rinsed in distilled water. They were prepared by dropping a few drops of cell suspension from arm's length. Three to four slides were prepared for each subject.

5. The slides were dried and aged at 37°C for three to five days for banding.

IV. G-Banding Technique

G-banding was done using the standard Giemsa-Trypsin-G-band method (Benn and Perle, 1986).

Procedure

1. The aged slides were dipped for 15-25 seconds in a Coplin jar containing chilled trypsin solution.

2. The slides were immediately rinsed in HBSS, followed by another rinse in HBSS.

3. The slides were stained with 10% Giemsa stain for 10-15 minutes, rinsed with distilled water, and air dried.

4. Air-dried slides were mounted with DPX and scanned for metaphases.

5. The slides were scanned for good, well-spread metaphases using a BX51 microscope (Olympus) and a GSL 10 system (Leica). Some metaphases were photographed using a CCD camera Zen 2012 (Zeiss, Germany). The karyotyping was done using CytoVision software (Leica Biosystems Richmond, Inc.) on a GSL-10 automated karyotyping system. 

6. Well-spread metaphases were analyzed, photographed, and then karyotyped.

7. The chromosomal aberrations observed were classified using the International System for Human Cytogenetic Nomenclature (ISCN, 2016).

Annexure III

Assessment of S-phase Fraction (SPF)

I. Preparation of Cell Suspension

The protocol given by Hemmer (1990) was followed with slight modifications as per laboratory conditions.

1. The tissue sample was put in a culture vial containing media and brought to the lab.

2. If adipose tissue was present, it was removed using a scalpel, and the tumor tissue was minced using a scalpel in a Petri dish.

3. The tissue was treated with collagenase and kept overnight at 370C. Fast pipetting was done without bubbling.

4. The cell suspension thus obtained was transferred to an autoclaved centrifuge tube, and the contents were centrifuged at 3000 rpm for 10 minutes.

5. The supernatant was discarded, and the pellet was resuspended in 5 ml of media. 1 ml of PBS was added to the centrifuge tube (covered with black paper).

6. Eighteen microliters of cytidine (final concentration 10 µM) and 1.8 µl of bromodeoxyuridine (final concentration 10 µM) were carefully added to the culture vial, which was then shaken gently.

7. Incubation of one hour was given at 37°C.

8. Contents were centrifuged at 3000 rpm for 10 minutes. The supernatant was discarded.

9. 70% ethanol was added to the pellet. The final volume was 8 ml, and soft pipetting was done. The tube containing the material was stored at -20°C until further processing.

II. Antibody Labeling

1. Ethanol-fixed cells were sedimented. 1 drop of pellet was put directly on a slide and air-dried.

2. DNA denaturation was done by adding a few drops of 2N HCL to the slide, which was incubated for 20 minutes. The slide was then washed in PBS.

3. Five microliters of anti-BrDU were added to the slide, which was incubated overnight in a humidified chamber. The slide was then washed with PBS. Excess PBS was carefully absorbed using filter tissue.

4. The procedure was then done in the dark. Ten microliters of DAPI (0.1 µg/ml), which acted as a counterstain, were put on the slide, and a coverslip was placed.

5. The slide was examined under the fluorescent microscope (Olympus BX51) using a 395 nm filter.

6. Cells in the S-phase gave green fluorescence. The number of cells in S-phase, divided by the total number of cells examined, gave the S-phase fraction.

7. The cells were photographed with a digital camera attached to the microscope at 1.6 msec exposure time.
